# A7׳ta: Data on a monolingual Arabic parallel corpus for grammar checking

**DOI:** 10.1016/j.dib.2018.11.146

**Published:** 2018-12-04

**Authors:** Nora Madi, Hend S. Al-Khalifa

**Affiliations:** Department of Information Technology, College of Computer & Information Sciences, King Saud University, Riyadh, Saudi Arabia

**Keywords:** Error checking, Arabic language, NLP, Parallel corpus

## Abstract

Grammar error correction can be considered as a “translation” problem, such that an erroneous sentence is “translated” into a correct version of the sentence in the same language. This can be accomplished by employing techniques like Statistical Machine Translation (SMT) or Neural Machine Translation (NMT). Producing models for SMT or NMT for the goal of grammar correction requires monolingual parallel corpora of a certain language.

This data article presents a monolingual parallel corpus of Arabic text called A7׳ta (). It contains 470 erroneous sentences and their 470 error-free counterparts. This is an Arabic parallel corpus that can be used as a linguistic resource for Arabic natural language processing (NLP) mainly to train sequence-to-sequence models for grammar checking. Sentences were manually collected from a book that has been prepared as a guide for correctly writing and using Arabic grammar and other linguistic features. Although there are a number of available Arabic corpora of errors and corrections [2] such as QALB [10] and Arabic Learner Corpus [11], the data we present in this article is an effort to increase the number of freely available Arabic corpora of errors and corrections by providing a detailed error specification and leveraging the work of language experts.

**Specifications table**

**Table:** 

Subject area	*Computer Science*
More specific subject area	*Computational linguistics, natural language processing, Linguistic Error Checking corpus. Arabic language.*
Type of data	*Text files, figures*
How data was acquired	*Corpus was manually extracted from the book (Linguistic Error Detector – Saudi Press as a Sample)**[1]*.
Data format	*Raw*
Experimental factors	*Texts are manually converted to text format.*
	*Texts are cleaned by removing extra spaces and unnecessary punctuation.*
	*Text files are divided into folders according to the book’s categorizations.*
Experimental features	*The data contains 300 documents, 470 erroneous sentences and their error-free counterparts, and a total of 3532 words.*
Data source location	*Saudi Arabia*
Data accessibility	*Data is with this article and can be downloaded from*https://github.com/iwan-rg

**Value of the data**

• Despite having QALB corpus [10] and the Arabic Learner Corpus [11], there still is a lack for monolingual parallel detailed specification data of erroneous/correct Arabic [2].
• This data could be used for training machine learning models (i.e. classifiers, neural networks, etc.) to recognize linguistic errors in Arabic text [3], [4].
• Extracted sentences can be used as samples in learning the Arabic language and some of its linguistic properties [1].
• This data can be used either as a parallel corpus or turned into an error-annotated corpus.

## 1 Data

The corpus is a collection of Modern Standard Arabic (MSA) sentences (and words) extracted from the book  (Linguistic Error Detector – Saudi Press as a Sample) [1]. The corpus contains about 470 erroneous sentences and their 470 error-free counterparts structured in text files. Each pair of sentences differs in one or more words. There are 300 documents and a total of 3532 words.

**Fig. 1:**
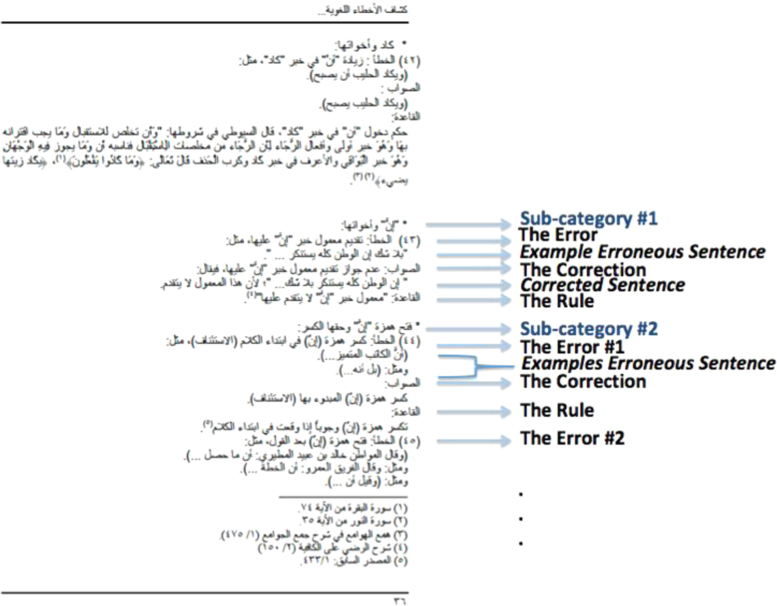
Sample page from "Linguistic Error Detector" book.

**Fig. 2:**
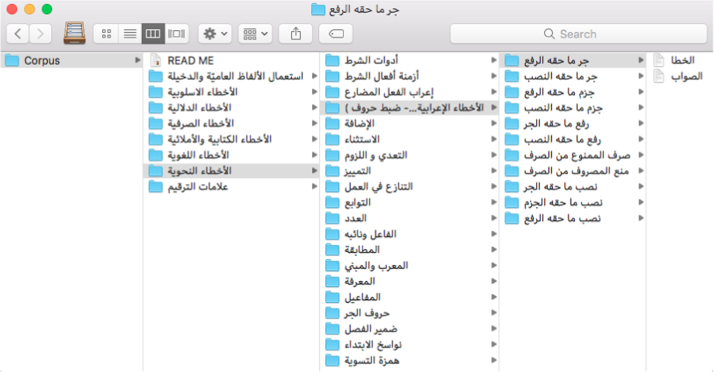
General corpus structure.

Data is presented in Fig. 1, Fig. 2, and at https://github.com/iwan-rg.

## 2 Experimental design, materials, and methods

The created corpus is based on a book called  (Linguistic Error Detector – Saudi Press as a Sample) [1]. One of the main goals of this book was to provide a simple guide for those who care about preserving and maintaining the proper form of written Arabic especially those in the fields of journalism and media in general. The book analyzed five Saudi newspapers: Al-Jazirah [5], Al Riyadh [6], Al Watan [7], Asharq Al-Awsat [8], and Al Shorouq [9]. Writing errors were manually mined from the newspapers and categorized into eight main categories: syntax errors, morphological errors, semantic errors, linguistic errors, stylistic errors, spelling errors, punctuation errors, and the use of informal as well as borrowed words. Each category of errors in the book contains sub-categories, which in turn consist of one or more specific types of errors. Further, each type of error is defined by three points (Fig. 1):

1. **The Error:** Defines what the error is, along with one or more examples from the newspaper articles.
2. **The Correction:** Defines how the correct version should be and corrects the examples listed in “The Error” point.
3. **The Rule(s):** Explains the linguistic rule(s) that this type of error adheres to.

Sentences were manually extracted from the electronic version of the book and organized into text files (Fig. 2). The extraction process:

1. We created a folder for each of the eight main categories.
2. Within each folder, we created a sub-folder for each sub-category within the main category if any.
3. Inside each main folder or sub-folder, folders were created for each type of error.
4. Within each error type folder, two files were created; one for the correctly written sentences () and another for the erroneous sentences ().

Since correct sentences were put in a separate file from the erroneous sentences, a model could be trained using only the correct sentences and another model could be trained on annotated erroneous sentences depending on the task. Also, since each pair of sentences differs in one or more words, it would be simple to compare each sentence in a pair and label each erroneous word.
